# HOXD8/DIAPH2-AS1 epigenetically regulates PAX3 and impairs HTR-8/SVneo cell function under hypoxia

**DOI:** 10.1042/BSR20182022

**Published:** 2019-01-25

**Authors:** Yaling Feng, Jianxia Wang, Yue He, Heng Zhang, Minhui Jiang, Dandan Cao, Aiping Wang

**Affiliations:** 1 Department of Perinatal Health Care, Wuxi Matemal and Child Health Hospital Affiliated to Nanjing Medical University, Wuxi, Jiangsu Province 214002, PR China; 2 Department of Women Health Care, Wuxi Matemal and Child Health Hospital Affiliated to Nanjing Medical University, Wuxi, Jiangsu Province 214002, PR China; 3 Department of Child Health Care, Wuxi Matemal and Child Health Hospital Affiliated to Nanjing Medical University, Wuxi, Jiangsu Province 214002, PR China

**Keywords:** DIAPH2-AS1, epigenetic regulation, HOXD8, PAX3

## Abstract

The present study aimed to unravel the molecular basis underlying PAX3 down-regulation, known to be involved in pre-eclampsia (PE) occurrence and development. Data obtained from databases suggested that *Pax*3 methylation levels in the promoter region are high in the placentas of PE patients. However, the expression of methylation-adjusting enzymes, including DNMT1, LSD1, and EZH2, did not change. Since lncRNAs enhance the function of methylation-related enzymes independently of expression, we selected three lncRNAs, RP11-269F21.2, DIAPH2-AS1, and RP11-445K13.2, predicted to interact with methylation-adjusting enzymes. Two transcription factors, HOXD8 and Lhx3, predicted to regulate the expression of lncRNAs, were also selected. Using RNA interference technology, HOXD8 and Lhx3 were found to positively regulate DIAPH2-AS1 and RP11-445K13.2 in HTR-8/SVneo cells. Chromatin immunoprecipitation assays determined that DIAPH2-AS1 recruited LSD1 to histone 3, increasing DNMT1 stability at H3. The HOXD8/DIAPH2-AS1 network regulated HTR-8/SVneo cell function under hypoxia by epigenetically regulating PAX3. This regulatory network may thus be responsible for PAX3 down-regulation in the placentas of PE patients.

## Introduction

Pre-eclampsia (PE) is a common obstetrical disorder with characteristic symptoms including hypertension and proteinuria. It has emerged as an important cause of maternal and perinatal morbidity and mortality for which there is currently no effective treatment except the termination of pregnancy [1]. The pathogenesis of PE is likely to be related to the impaired development of the placenta, which consequently causes a series of pathological processes, such as endothelial dysfunction, systemic inflammation, and oxidative stress [2]. Our previous study identified that abnormal down-regulation of PAX3 expression plays a critical role in the occurrence and development of PE. Trophoblasts with a low PAX3 expression show inhibited proliferative and invasive capacities [3], whereas the invasion of the foetally derived extravillous trophoblasts (EVTs) toward the myometrium layer of the uterine wall is a key step in placenta development [4,5]. An impaired invasion of the trophoblasts is likely to cause defective arterial remodeling, resulting in inadequate blood flow in the placenta [6].

The expression of most genes is generally regulated by complex molecular mechanisms involving many factors, including transcription factors, long non-coding RNAs, and enzymes regulating the methylation of genes and histones. Among these, long non-coding RNAs (lncRNAs), a new member of the non-coding RNA family (ncRNA), can function as signals, decoys, guides, or scaffolds to participate in intertwined gene regulatory networks at various levels [7]. LncRNAs harbor many miRNA response elements and therefore can communicate and regulate other RNA transcripts by competing specifically for shared miRNAs. Moreover, mounting evidence have shown that some lncRNAs can coordinate histone modifications by binding to various histone modification enzymes, such as lysine-specific demethylase 1 (LSD1) and enhancer of zeste homolog 2 (EZH2). LSD1 demethylates mono- and di-methylated residues of lysine-4 on histone H3 (H3K4me1, H3K4me2, and H3K9me1) and results in transcriptional repression, but LSD1 also activates transcription through the demethylation of H3K9me2 [8]. In addition to histone 3, LSD1 also demethylates and thus stabilizes DNA methyl-transferase 1 (DNMT1). Targeted deletion of the gene encoding LSD1 in embryonic stem cells correlates with decreased DNMT1, resulting in progressive loss of DNA methylation [9]. These data indicate that LSD1 also regulates DNA methylation [9]. As such, LSD1 is likely to mediate lncRNAs, epigenetically regulating gene expression by influencing DNA methylation.

Previous studies have used high-throughput genomic and epigenomic technologies to investigate the difference in gene expression at various transcriptional levels between patients with PE and healthy participants. The data from the GSE44667 database showed high methylation levels of the *PAX3* gene in the placenta of patients with PE [10]. This result is consistent with the low expression of *PAX3* in the same patients, which was identified in our previous study [3]. Therefore, this evidence suggest that high methylation levels of *PAX3* gene are an important cause of low expression. The present study aimed to elucidate the mechanism underlying the abnormally high methylation levels of *PAX3* gene in the placenta of patients with PE.

## Materials and methods

### Sample collection

Placentas (PE, *n*=10 and normal, *n*=10) were collected from women who had undergone Caesarean deliveries between January 2015 and December 2015 at the Department of Gynaecology and Obstetrics in the Wuxi Maternity and Children Health Hospital Affiliated Nanjing Medical University (Wuxi, Jiangsu, China). All protocols used in the present study were approved by the Research Medical Ethics Committee of Wuxi Maternity and Children Health Hospital and the study abides by the Declaration of Helsinki principles. All women were informed of the research nature of our study and signed informed consent forms. The clinical data from all patients and normal controls are recorded in Table 1.

**Table 1:** Clinical information of healthy controls and PE patients

	Control (*N*=10)	PE (*N*=10)	*P*-value
Age (year)	30.5 ± 4.37	31.3 ± 4.84	0.063
Gestational age (week)	39.6 ± 5.42	37.2 ± 4.93	0.041
SBP (mmHg)	116.7 ± 20.92	171.5 ± 29.41	<0.01
DBP (mmHg)	77.6 ± 13.78	103.8 ± 19.6	0.025
Proteinuria (g/24 h)	N/A	3.27±0.59	N/A

Abbreviations: DBP, diastolic pressure; SBP, systolic pressure.

### Cell culture and treatments

HTR-8/SVneo cells were obtained from ATCC (American Type Culture Collection, Manassas, VA) and maintained in RPMI-1640 medium (Life Technologies, Carlsbad, CA, U.S.A.) supplemented with 10% foetal bovine serum (Invitrogen, Carlsbad, CA, U.S.A.), and antibiotics (100 U/ml penicillin and 100 μg/ml streptomycin). For regular maintenance, the cells were grown in 75-cm^2^ plastic flasks at 37°C in a 5% CO_2_-95% air atmosphere with media changes every 2–3 days. For hypoxia, the cells were cultured in an AW200SG hypoxic workstation (Electrotek, U.K.) using a continuous flow of a humidified mixture of 1% O_2_, 5% CO_2_, and 94% N_2_ at 37°C. Experiments were performed while the cells were under either hypoxic (2% O_2_) or normoxic (20% O_2_, as a control) conditions.

### Cell transfection

HTR8/SVneo cells were transfected with various siRNAs designed by GenePharma (Shanghai, China) to knockdown the targeted genes. Table 2 shows the siRNA sequences. HTR8/SVneo cells were seeded onto six-well plates with a density of 1 × 10^5^ cells/well. The cells were transfected with these siRNAs using Lipofectamine RNAi Max (Thermo Fisher Scientific, Waltham, Massachusetts, U.S.A.), according to the manufacturer’s instructions. For the ectopic expression of Pax3 in HTR-8/SVneo cells under hypoxia, transfection of pEGFP-C1-Pax3 vector (Genephama Biotech) was performed using the Lipofectamine 2000 of Thermo Fisher Scientific, according to the manufacturer’s instructions. Cell viability, migration, and invasion assays were performed 48 h after the transfection.

**Table 2:** The sequences of siRNAs

Name	Sense (5′-3′)	Antisense (5′-3′)
HOXD8-SiRNA-702	UAGUAAGUGGGAUUGAUGGTT	CCAUCAAUCCCACUUACUATT
HOXD8-SiRNA-1210	UCUUCCUCUUCGUCUACCATT	UGGUAGACGAAGAGGAAGATT
HOXD8-SiRNA-1534	AGCAUAUGGUGAUUAUUAGTT	CUAAUAAUCACCAUAUGCUTT
LHX3-SiRNA-823	UCAUGUUGCGGAAAUACUGTT	CAGUAUUUCCGCAACAUGATT
LHX3-SiRNA-1625	UUUCAUGUCUAGAAAUAGCTT	GCUAUUUCUAGACAUGAAATT
LHX3-SiRNA-2312	AAUAGGUAGCUCGAGAUUCTT	GAAUCUCGAGCUACCUAUUTT
DIAPH2-AS1-SiRNA-196	AGUUGUUAUGUCUUCUUAGTT	CUAAGAAGACAUAACAACUTT
DIAPH2-AS1-SiRNA-341	AGGAUAAUCGCUUGAAUCCTT	GGAUUCAAGCGAUUAUCCUTT
DIAPH2-AS1-SiRNA-867	UAGGUUAAUUCCAUUUAUGTT	CAUAAAUGGAAUUAACCUATT
RP11-445K13.2-SiRNA-202	AUAAAGCAAUGUCGUUAUGTT	CAUAACGACAUUGCUUUAUTT
RP11-445K13.2-SiRNA-252	UCUCAAAGAGAAUCAGUAGTT	CUACUGAUUCUCUUUGAGATT
RP11-445K13.2-SiRNA-338	UUUGUAACUGGCCAAUUUCTT	GAAAUUGGCCAGUUACAAATT
Negative control FAM	UUCUCCGAACGUGUCACGUTT	ACGUGACACGUUCGGAGAATT

### Real-time quantitative PCR

Total RNA was extracted using a mirVana miRNA isolation kit (Thermo Fisher Scientific). A High Capacity RNA-to-cDNA Master Mix (Life Technologies) was used to synthesize cDNA. Real-time quantitative PCR was performed using the SYBR ExScript RT-PCR kit (TaKaRa, Dalian, China) on an ABI 7300 Real-Time PCR System (Applied Biosystems, Foster City, CA, U.S.A.) according to manufacturer’s instructions. The thermocycling profiles were as follow: 95°C for 30 s, followed by 40 cycles of 95°C for 5 s and 60°C for 30 s. The sequences of the primers are shown in Table 3.

**Table 3:** Primer sequences in PCR assay

Gene name	Direction	Sequence (5′–3′)	Length (bp)	TM (°C)
GAPDH	Forward	GGAGCGAGATCCCTCCAAAAT	197	61
	Reverse	GGCTGTTGTCATACTTCTCATGG		
RP11-269F21.2	Forward	AGGTTCCAAACCAGGAGCT	300	60
	Reverse	CCCTTCTACCTTGGCAGTGA		
DIAPH2-AS1	Forward	GGATCTTGCATTGGAGGAGA	166	60.5
	Reverse	ATGGTGGCACGTGTCTGTA		
RP11-445K13.2	Forward	GCCCCAGAGAGATATGTCCA	188	60
	Reverse	TGAAGGCACTGCAATCATGT		
				

### Western blot analysis

Frozen placenta tissue and cell samples were homogenized and lysed in RIPA lysis buffer (Beyotime, Shanghai, China) supplemented with phenylmethanesulfonyl fluoride (PMSF, Beyotime). The protein concentration of the supernatant was quantified using the BCA Reagent Kit (Beyotime). Equal amounts of the protein samples (20 μg) were separated by electrophoresis on a 10 or 12% sodium dodecyl sulphate–polyacrylamide gel and transferred to a polyvinylidene fluoride (PVDF) membrane (Bio-Rad, CA, U.S.A.). After blocking with non-fat dry milk (Yili Milk company, Inner Mongolia, China) at room temperature for 2 h, the membranes were hybridized with anti-Pax3 antibody (Dilution 1:1000, ab180754, Abcam, Cambridge, U.K.), anti-HOXD8 antibody (Dilution 1:200, ab229321, Abcam), anti-LHX3 antibody (Dilution 1:400, ab124697, Abcam), and anti-GAPDH antibody (Dilution 1:2000, SC-365062, Santa Cruz Biotechnology, Santa Cruz, CA, USA) at 4°C overnight. The primary antibodies were visualized by adding secondary biotin-conjugated antibodies followed by an avidin/biotin/peroxidase complex (Vectastain ABC Elite kit; Vector Laboratories lnc, Burlingame, CA, U.S.A.) and substrate (Vector NovaRED, Vectastain). Quantification was performed by densitometric analysis using Quantity One software (Bio-Rad).

### Luciferase reporter assay

Bioinformatics analysis by Alggen identified six putative binding sites of HOXD8 on the promoter (within 1000bp) of *DIAPH2-AS1* gene. A wild-type sequence (-100 ∼ -900 bp) containing all these binding sites was established by PCR. In addition, two of the binding sites (5′-ATAAAAC-3′ and 5′-AATTTAT-3′) with the highest scores in the bioinformatics analysis were mutated in the wild-type sequence to established two mutant sequences. The wild-type and mutant sequences were cloned into the pGL3 vector. The vectors were transfected into HTR8/SVneo cells alone or with HOXD8 over-expression vector using Lipofectamine 2000 (Invitrogen). Cells were harvested at 24 h and the activity of firefly luciferase was normalized to that of renilla luciferase.

### Chromatin immunoprecipitation assay

The EZ-Magna ChIP kit (EMD Millipore) was used to conduct the chromatin immunoprecipitation (ChIP) assays in accordance with the manufacturer’s protocol. After carrying out the above-mentioned cell treatments, the cells were lysed with Cell Lysis Buffer and Nuclear Lysis Buffer and sonicated to obtain chromatin fragments. Next, the lysates were immunoprecipitated with Magnetic Protein A Beads conjugated with Histone3 (Millipore) or IgG (Millipore) as a control. Western blot assay was performed to detect the enrichment of LSD1, H3K27me1 (Millipore), H3K4me2 (Millipore), and DNMT1 (Millipore) in the precipitated protein complex.

### RNA immunoprecipitation

RNA immunoprecipitation (RIP) was performed on HTR8/SVneo cells using EZ-Magna RIP™ RNA-Binding Protein Immunoprecipitation Kit (Millipore) to investigate interactions between LSD1 and DIAPH2-AS1. Briefly, HTR8/SVneo cells were lysed with RIPA-2 buffer and then incubated with Protein-A Dynabeads conjugated with IgG or LSD1 antibodies. The bead/antibody/lysate mixture was incubated at 4°C overnight rotating end-over-end. Beads were washed with cold NT2 buffer five times. Proteinase K treatment released RNAs from bound proteins and input and bound RNA was isolated with TRIzol (Invitrogen) and reverse transcribed as described above. Enrichment for DIAPH2-AS1 with LSD1 immunoprecipitation was calculated using the comparative *C*_t_ method, with samples normalized to input and compared to IgG control. Data are presented as fold enrichment relative to GAPDH enrichment for each sample.

### Methylated DNA immunoprecipitation-PCR

Methylated DNA immunoprecipitation (MeDIP) is a large scale antibody-based technique that is used to enrich and capture methylated DNA fragments for use in gene-specific DNA methylation studies on a genome wide scale. MeDIP was performed in HTR8/SVneo cells using the EpiQuik™ Methylated DNA Immunoprecipitation Kit (Epigentek, Wuhan, China). DNA in the cells was extracted, sheared, and added into the microwell immobilized with the ChIP-grade 5-methylcytosine antibody. DNA is released from the antibody–DNA complex, and purified through the specifically designed Fast-Spin Column. Eluted DNA can be used for PCR tests. The methylated-specific primer for the promoter region of Pax3 was designed using MethPrimer (http://www.urogene.org/methprimer/). The primer was sense: 5′-ATTTTTGAGAATAAACGTTGAATTC-3′ plus antisense: 5′-ATAAAAACCGCTCTAACAAACTACG-3′. PCR was performed according above-mentioned method.

### Proliferation assay

HTR-8/SVneo cells were seeded in triplicate at 1 × 10^3^ cells/well onto 96-well microplates. After the above-mentioned cell treatments, 20 μl (at 5 mg/ml) of 3-(4,5-dimethyl-2-thiazolyl)-2,5-diphenyl-2H-tetrazolium bromide (MTT; Sigma, St. Louis, MO, U.S.A.) was added to each well. The medium containing MTT was removed after 4 h and 150 μl of dimethylsulfoxide was added subsequently to terminate the reaction. A microplate reader was used to examine absorbance of each well of the plate at 570 nm.

### Scratch assay

HTR-8/SVneo cells were seeded onto six-well plates and subjected to the indicated treatments. A 1-ml pipette tip was used to scratch a line on the cell monolayer. The culture medium was replaced with FBS-free medium in the following cell culture under the normoxia or hypoxia for 24 h. Microscopic images of the same area were captured immediately after the scratching as well as after 48 h. The cell migration rate was calculated based the formula: cell migration rate (%) = (initial distance − final distance) / initial distance ×100%.

### Transwell cell invasion assay

HTR-8/SVneo cells were seeded in the upper chamber of Transwell chambers (8 μm pore size; Corning; Corning, NY, U.S.A.). The complete medium was added to the lower chamber. After culturing under normoxia or hypoxia for 48 h, non-invading cells on the upper surface of the membrane were removed with a cotton-tipped swab, while the invading cells on the bottom surface were fixed with ice-cold methanol for 30 min and stained with 0.1% crystal violet. The invading cells were quantified by counting ten random fields at ×200 magnification.

### Statistical analysis

Data were analysed using SPSS 12.0 software (SPSS, Inc., Chicago, IL, U.S.A.). The statistical significances of the differences were evaluated by performing one-way analysis of variance (one-way ANOVA) followed by the Scheffe’s post-hoc test. *P*<0.05 was considered to indicate a statistically significant difference.

## Results

### The analysis of databases

**Figure 1:**
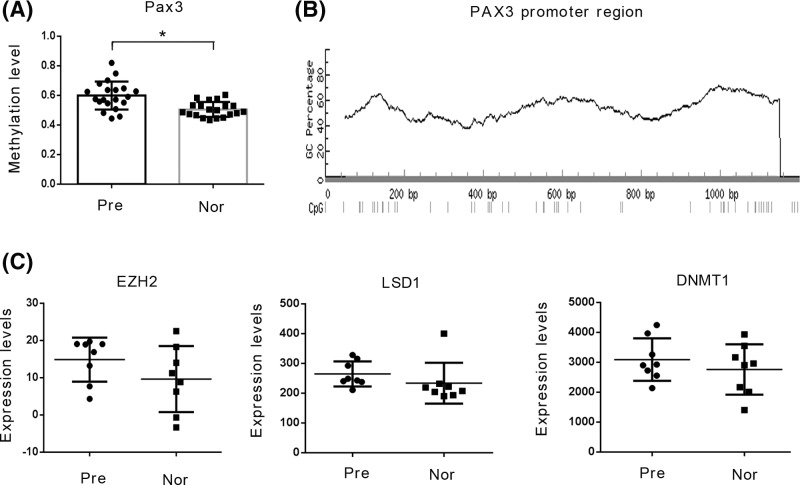
Expression profiles of PAX3, DNMT1, LSD1, and EZH2 in databases (**A**) The methylation level of the *Pax*3 gene in the promoter region was based on the data from the GSE44667 database. (**B**)The methylation sites at the promoter region of *Pax*3 gene were predicted by MethPremer 2.0 (http://www.urogene.org/methprimer/). (**C**) The mRNA expression of DNMT1, LSD1, and EZH2 was based on the data from the GSE44711 database. *P<0.05.

As indicated by the data from the GSE44667 database [10], the methylation levels of the *Pax*3 gene in the promoter region (1200 bp) are higher in the placenta tissue of PE patients compared with those of healthy controls (*P*<0.05, Figure 1A). Figure 1B shows the methylation distribution in the promoter region. EZH2, LSD1, and DNMT1 constitute a series of enzymes responsible for regulating the methylation of genes and histones. However, according to data from the GSE44711 database [11], there was no significant difference in the expression of these enzymes in the placenta tissue between patients with PE and the healthy controls (Figure 1C). Previous works have reported that lncRNAs enhance the function of methylation-related enzymes independently of their expression. This effect is likely related to lncRNAs recruiting the enzymes to their substrates. By analyzing the data from the GSE50783 database, we found that 16 lncRNAs were significantly up-regulated in the placenta tissue of the PE patients (Table 4). Among the up-regulated lncRNAs, RP11-269F21.2, DIAPH2-AS1, and RP11-445K13.2 were predicted to bind to EZH2 and/or LSD1 by bioinformatics analysis using RNA–Protein Interaction Prediction (RPISeq; http://pridb.gdcb.iastate.edu/RPISeq/). The up-regulation of these lncRNAs is likely due to the enhanced function of transcription factors. The data from the GSE50783 [12] database also showed 12 up-regulated transcription factors in the placenta tissue of PE patients (Table 5). Among the up-regulated transcription factors, HOXD8 and Lhx3, were predicted to regulate the expression of RP11-269F21.2, DIAPH2-AS1, and RP11-445K13.2 by bioinformatics analysis using Alggen (http://alggen.lsi.upc.es/) (Table 6) and Jaspar (http://jaspar2016.genereg.net/) (Table 7). Based on all these analyses, we hypothesized that the increase of *Pax*3 gene methylation is associated with a complex regulatory network related to transcription factors, lncRNAs, and methylation-adjusting enzymes.

**Table 4:** GSE50783 database shows 16 up-regulated lncRNAs in the placenta tissue of PE patients compared with those of healthy controls

Gene symbol	Sequence name	Up-regulaton (fold change)
**RP11-269F21.2**	**ENST00000508010**	**3.6478987**
PSG10	NR_026824	2.624768
lincRNA-RDH13	NR_027382	2.2103374
**lincRNA-DIAPH2-1**	**DIAPH2-AS1**	**2.09231**
RHOBTB1	NR_024555	2.0465794
ZIC4	NR_033118	1.9706011
ZIC4	NR_033119	1.9173626
RP4-555L14.5	ENST00000489632	1.9004802
lincRNA-JAK2-1	BX098775	1.8901271
	AK128878	1.8824089
RP11-265F19.1	ENST00000506476	1.815456
	AK124889	1.7879285
BC020168	uc003tig.1	1.7773525
LOC100128788	NR_027275	1.6350112
HMGB3L1	NR_002165	1.5528641
**RP11-445K13.2**	**ENST00000448272**	**1.5394561**

The data were from five normal placenta tissue samples and six PE placenta tissue samples.

Terms in bold signify *P* <0.05

**Table 5:** GSE50783 database shows 12 up-regulated transcription factors in the placenta tissue of PE patients compared with those of healthy controls

Gene symbol	Transcription factor	Up-regulaton (fold change)
**NM_019558**	**HOXD8**	**2.795836**
NM_001082578	RBM9༌RBFOX2	2.6311963
NM_003670	BHLHE40	2.4996438
NM_012289	KEAP1	2.4178288
**NM_014564**	**LHX3**	**2.344015**
TBS; HSAL1;	SALL1	1.814546
MSG1	CITED1	1.8138658
FKLF; FKLF1;	KLF11	1.7985306
GTAR;	ANKRD17	1.7296797
CMD1J; DFNA10	EYA4	1.7221911
EBAF; LEFTA; TGFB4; LEFTYA	LEFTY2	1.622051
NM_001421	ELF4	1.5281811

The data were from five normal placenta tissue samples and six PE placenta tissue samples.

Terms in bold signify *P* <0.05

**Table 6:** The predicted binding sites of HOXD8 at promoter region of genes

Name	Start position	End position	Binding site sequences
RP11-269F21.1	733	739	ATAATAG
RP11-269F21.1	759	765	ATAATTA
RP11-269F21.1	965	971	AGTTTAT
DIAPH2-AS1	117	123	ATAAATA
DIAPH2-AS1	248	254	GTATTAT
DIAPH2-AS1	253	259	ATAAAAC
DIAPH2-AS1	308	314	ATAAGAT
DIAPH2-AS1	862	868	AATTTAT
RP11-445K13.2	340	346	CCTTTAT
RP11-445K13.2	519	525	ATAAGCT
RP11-445K13.2	526	532	TGCTTAT
RP11-445K13.2	627	633	ATAAAGA
RP11-445K13.2	656	662	ATAAAGC

The data were from Alggen (http://alggen.lsi.upc.es/).

**Table 7:** The predicted binding sites of Lhx3 at promoter region of genes

Factor name	Start position	End position	Binding site sequences
RP11-269F21.1	757	769	GAAATAATTAATT
RP11-269F21.1	761	773	TAATTAATTCCTG
DIAPH2-AS1	293	305	AAATTAAAAAGCT
DIAPH2-AS1	875	887	AACTTAATTCTTA
RP11-445K13.2	370	382	AAATTAACCATCA

The data were from Jaspar (http://jaspar2016.genereg.net/).

### Expression profile of indicated genes and proteins in placenta tissue

**Figure 2:**
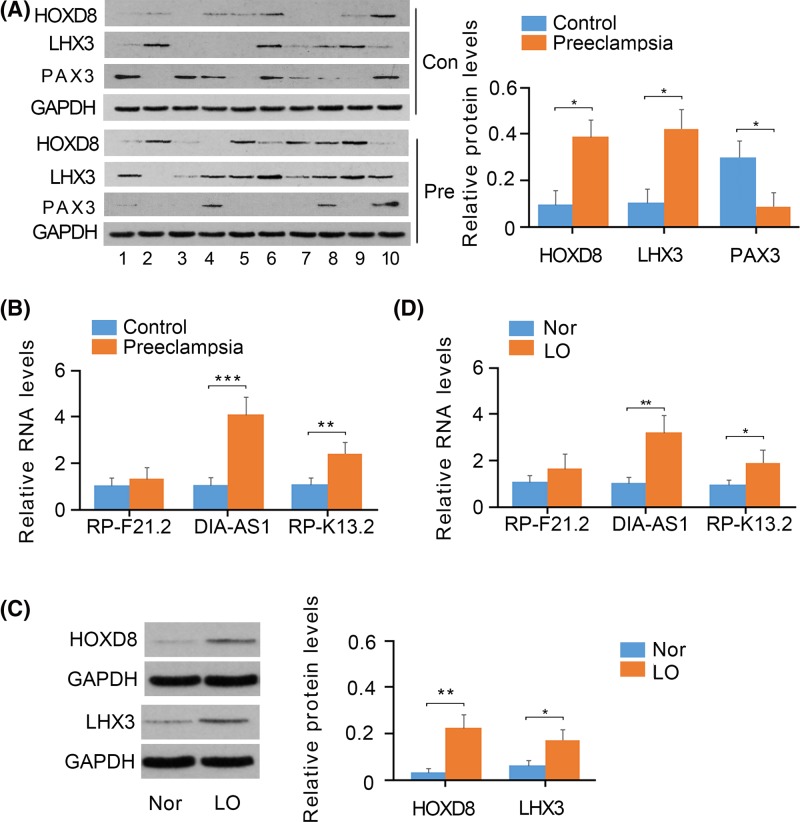
Expression of transcription factors and lncRNAs in tissues and cells (**A**) HOXD8, Lhx3, and Pax3 expression in the placenta tissues of both PE patients and healthy controls was measured using Western blot assays. (**B**) RP11-269F21.2, DIAPH2-AS1, and RP11-445K13.2 expression in the placenta tissue of PE patients and healthy controls was measured using PCR assays. (**C**) HOXD8 and Lhx3 expression in HTR-8/SVneo cells under normoxia and hypoxia was measured using Western blot assays. (**D**) RP11-269F21.2, DIAPH2-AS1, and RP11-445K13.2 expression in HTR-8/SVneo cells under normoxia and hypoxia was measured using PCR assays. *P<0.05, **P<0.01, and ***P<0.001. Nor: normoxia; LO: Low oxygen (hypoxia).

To verify the data obtained from databases, we detected the expression of the above-selected genes and proteins in the placenta tissue of both PE patients and healthy participants. We collected ten placenta tissue samples from PE patients and another ten age-matched placenta tissue samples from healthy participants. The protein levels of both HOXD8 and Lhx3 were found to be increased in PE patients compared with that in healthy participants (*P*<0.05, Figure 2A), while Pax3 protein levels were decreased in patients with PE (*P*<0.05). As indicated by PCR, the expression of DIAPH2-AS1 (*P*<0.001, Figure 2B) and RP11-445K13.2 (*P*<0.01, Figure 2A), but not RP11-269F21.2, was higher in PE patients.

### Expression profile of indicated genes and proteins in HTR-8/SVneo cells under hypoxic conditions

The cultivation of HTR-8/SVneo cells under hypoxic conditions is a classical cell model for imitating the pathological state of trophoblast cells in patients with PE, considering the defective arterial remodeling in the patients results in inadequate perfuse of blood flow in the placenta. Hypoxia caused an increase in the protein levels of HOXD8 (*P*<0.01, Figure 2C) and Lhx3 (*P*<0.05) in HTR-8/SVneo cells. Moreover, the increase was greater for HOXD8 than Lhx3. PCR analysis showed that the expression of DIAPH2-AS1 (*P*<0.01, Figure 2D) and RP11-445K13.2 (*P*<0.05) also increased in HTR-8/SVneo cells under hypoxic conditions, with a greater increase in HOXD8.

### Knockdown of HOXD8 and Lhx3 reduced DIAPH2-AS1 and RP11-445K13.2 expression in HTR-8/SVneo cells under hypoxia

**Figure 3:**
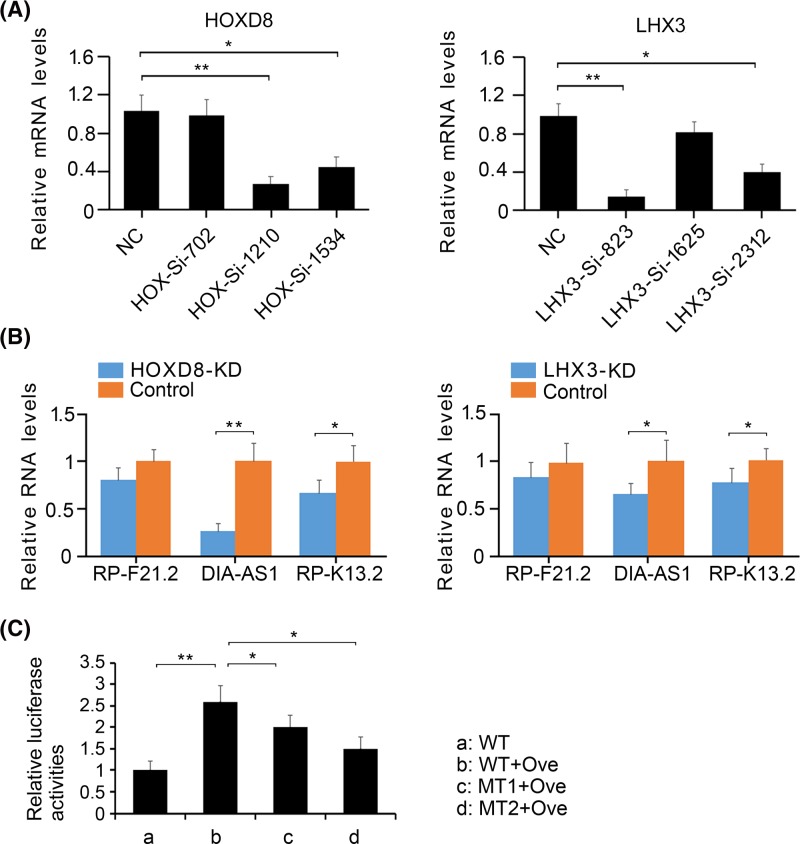
The regulatory effect of HOXD8 and Lhx3 on RP11-269F21.2, DIAPH2-AS1, and RP11-445K13.2 (**A**) HOXD8 and Lhx3 were knocked down in HTR-8/SVneo cells under hypoxia using RNA interference technology. (**B**) The expression of RP11-269F21.2, DIAPH2-AS1, and RP11-445K13.2 expression in HTR-8/SVneo cells under hypoxia was measured by PCR assay after HOXD8 and Lhx3 knockdown. (**C**) A wild-type sequence (-100 ∼ -900 bp) containing all putative binding sites of HOXD8 on the promoter of *DIAPH2-AS1* gene was cloned into pGL3 luciferase report. Two of the binding sites were mutated to established two mutant vectors. The vectors were transfected into HTR8/SVneo cells alone or with HOXD8 over-expression vector. *P<0.05 and **P<0.01. WT: wild-type vector; MT: mutant-type vector; Ove: HOXD8 overexpression vector. KD: knockdown

To determine whether the up-regulation of DIAPH2-AS1 and RP11-445K13.2 is associated with the actions of HOXD8 and Lhx3, we knocked down these transcript factors in HTR-8/SVneo cells under hypoxic conditions. As indicated by PCR analysis, HOXD8-SiRNA-1210 and LHX3-SiRNA-823 exerted the most profound effect on the inhibition of HOXD8 (*P*<0.01, Figure 3A) and Lhx3 (*P*<0.01), respectively. HOXD8-SiRNA-1210 and LHX3-SiRNA-823 were, thus, used for further study. The depletion of HOXD8 reduced the expression of DIAPH2-AS1 (*P*<0.01, Figure 3B) and RP11-445K13.2 (*P*<0.05) in HTR-8/SVneo cells under hypoxic conditions. Similar results were also observed after Lhx3 knockdown (*P*<0.05). Given the profound effect of HOXD8 on the regulation of DIAPH2-AS1 expression, we performed luciferase report assay to determine that HOXD8 regulating DIAPH2-AS1 is through directly binding to the promoter region. Overexpression of HOXD8 enhanced luciferase activity of the wild-type vectors (*P*<0.01, Figure 3C), and the promoting effect is much stronger on those of two mutant-type vectors (*P*<0.05) although they activities were also enhanced with HOXD8 overexpression.

### Knockdown of DIAPH2-AS1 and RP11-445K13.2 reversed *Pax3* expression in HTR-8/SVneo cells under hypoxia

**Figure 4:**
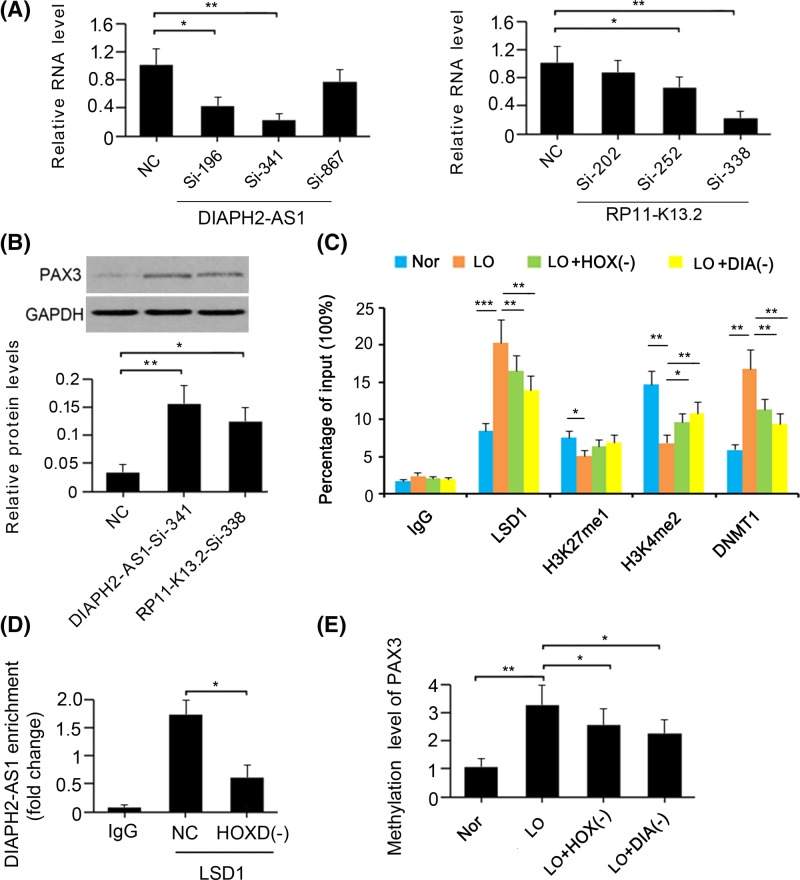
The regulatory effect of the HOXD8/DIAPH2-AS1 network on PAX3 expression (**A**) DIAPH2-AS1 and RP11-445K13.2 were knocked down in HTR-8/SVneo cells under hypoxia using RNA interference technology. (**B**) The expression of PAX3 in HTR-8/SVneo cells under hypoxia was measured by Western blot assay after DIAPH2-AS1 and RP11-445K13.2 knockdown. (**C**) The combination of LSD1 and DNMT1 with histone 3 was analyzed by chromatin immunoprecipitation assay after HOXD8 and DIAPH2-AS1 knockdown. (**D**) RIP assay was performed on HTR8/SVneo cells to investigate interactions between LSD1 and DIAPH2-AS1. The enrichment of DIAPH2-AS1 on LSD1 was determined by PCR after HOXD8 knockdown or not. (**E**) MeDIP-PCR assay was performed to evaluate the methylation levels at the promoter region of *Pax3* gene after the knockdown of HOXD8 and DIAPH2-AS1. *P< 0.05, and **P< 0.01. Nor: normoxia; LO: Low oxygen (hypoxia); HOX(-): HOXD8 knockdown; DIA(-):DIAPH2-AS1 knockdown.

To determine whether the down-regulation of Pax3 is associated with the actions of DIAPH2-AS1 and RP11-445K13.2, we knocked down these LncRNAs in HTR-8/SVneo cells under hypoxic conditions. PCR analysis showed that DIAPH2-AS1-SiRNA-341 and RP11-445K13.2-SiRNA-338 conferred the most profound effect on the inhibition of DIAPH2-AS1 (*P*<0.01, Figure 4A) and RP11-445K13.2 (*P*<0.01) expression, respectively. The inhibition of DIAPH2-AS1 and RP11-445K13.2 expression caused a notable increase in Pax3 protein levels (*P*<0.01 and *P*<0.05, respectively; Figure 4B). These results suggest that the expression of Pax3 in HTR-8/SVneo cells under hypoxia was mainly influenced by the HOXD8/DIAPH2-AS1 network. As such, we performed a ChIP assay to elucidate how this network modulates *Pax3* expression. The results suggested that hypoxia increased the accumulation of LSD1 (*P*<0.001; Figure 4C) and DNMT1 in histone 3(H3) (*P*<0.01). Moreover, increased LSD1 is associated with the reduced percentages of H3K27me1 (*P*<0.05) and H3K4me2 (*P*<0.01) in H3. However, the depletion of both HOXD8 and DIAPH2-AS1 hindered the accumulation of LSD1 (*P*<0.01) and DNMT1 (*P*<0.01) in H3, which correlated with an increased percentage of H3K4me2 (*P*<0.05 or *P*<0.01). RIP was performed to further determine the interaction between LSD1 and DIAPH2-AS1. DIAPH2-AS1 was identified to bind to LSD1, but the enrichment of DIAPH2-AS1 in LSD1 was decreased with HOXD8 knockdown (*P*<0.05, Figure 4D). MeDIP-PCR was performed to determine the effect of HOXD8/DIAPH2-AS1 cascades on the methylation at the promoter region of *PAX3* gene. The methylation level at the promoter region was increased under hypoxia (*P*<0.01, Figure 4E); however, either knockdown of HOXD8 or DIAPH2-AS1 inhibited the increase of the methylation level under hypoxia (*P*<0.05 vs. hypo group).

### The regulatory effect of the HOXD8/DIAPH2-AS1/Pax3 network on the proliferation and migration of HTR-8/SVneo cells under hypoxia

**Figure 5:**
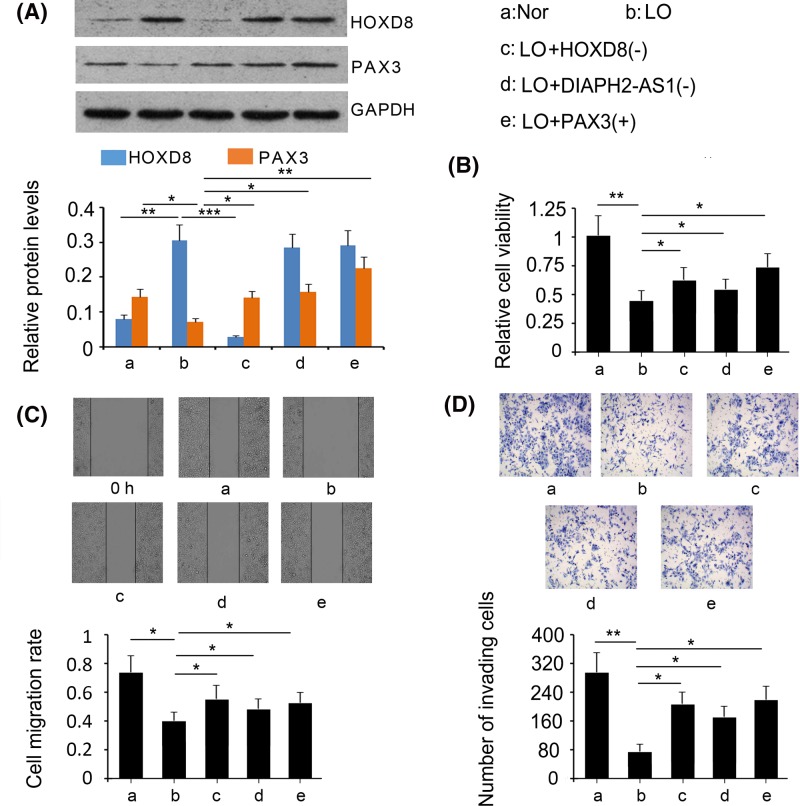
The regulatory effect of the HOXD8/DIAPH2-AS1/PAX3 network on cell proliferation and invasion Either HOXD8 or DIAPH2-AS1 were knocked down or PAX3 was overexpressed in HTR-8/SVneo cells under hypoxia. Afterward, HOXD8 and PAX3 expression was measured by Western blot assay (**A**). Cell viability was assessed using MTT testing agent (**B**). Cell migration and invasive capacities were evaluated by scratch (**C**) and transwell cell invasion (**D**) assays, respectively. *P<0.05, **P<0.01, and ***P<0.001. Nor: normoxia; LO: Low oxygen (hypoxia); HOX(-): HOXD8 knockdown; DIA(-):DIAPH2-AS1 knockdown; Pax3(+):Pax3 overexpression.

As indicated by Western blot assays, siRNA-mediated gene knockdown reversed the increase in HOXD8 expression in HTR-8/SVneo cells under hypoxia (*P*<0.001 vs. hypo group; Figure 5A). However, DIAPH2-AS1 knockdown and Pax3 overexpression did not affect the expression of HOXD8 in HTR-8/SVneo cells under hypoxia. The reduction of *Pax3* expression induced by hypoxia was reversed by either HOXD8 depletion (*P*<0.05 vs. hypo group), DIAPH2-AS1 knockdown (*P*<0.05 vs. hypo group), or *Pax3* overexpression (*P*<0.01 vs. hypo group). Inhibitory cell viability under hypoxia was partly restored by either HOXD8 depletion (*P*<0.05 vs. hypo group; Figure 5B), DIAPH2-AS1 knockdown (*P*<0.05 vs. hypo group), or *Pax3* overexpression (*P*<0.05 vs. hypo group). In addition, HOXD8 and DIAPH2-AS1 knockdowns, as well as *Pax3* overexpression, partly recovered the migratory and invasive capacities of HTR-8/SVneo cells under hypoxia (*P*<0.05 vs. hypo group; Figure 5C,D).

## Discussion

PAX3 functions as a transcription factor and plays an important role in the development of muscles, nerves, and the placenta. However, PAX3 is abnormally down-regulated in the placenta of PE patients, and a low expression of PAX3 is associated with impaired proliferation and invasion of trophoblast cells under hypoxia. This suggests that decreased expression of PAX3 is closely related to PE pathogenesis [3]. Previous studies have used high-throughput genomic and epigenomic technologies to identify higher methylation levels in the promoter region of *PAX3* gene in the placenta of PE patients, which has provided an important clue for elucidating the down-regulation mechanism of *PAX3*. Interestingly, the expression of enzymes (e.g. DNMT1, EZH2, and LSD1) responsible for regulating the methylation of genes and histones are not significantly changed in the placenta of PE patients compared with that of the healthy controls. However, the fact that the expression of these enzymes is unchanged does not mean that their functions are not changed. Previous works have reported that lncRNAs can enhance the function of methylation-related enzymes independently of changing their expression. For example, some lncRNAs, such as HOXA-AS2, LINC00673, and LincRNAFEZF1-AS1, recruit and bind with LSD1, which enhances demethylation function atH3, thereby influencing chromatin compression tightness in suppressing gene expression [13,14]. In this process, lncRNAs function as a scaffold recruiting this methylation-adjusting enzyme to its subjects. Therefore, this change in the location of the enzyme in cells greatly affects its functions.

In the present study, lncRNAs including RP11-269F21.2, DIAPH2-AS1, and RP11-445K13.2 were selected from previous databases due to their up-regulation in the placenta of PE patients and to their putative interactions with LSD1 and/or EZH2, as indicated by bioinformatics analysis. In addition, transcription factors HOXD8 and Lhx3 were also selected from previous databases as they may be responsible for the up-regulation of these lncRNAs. The up-regulation of DIAPH2-AS1, RP11-445K13.2, HOXD8, and Lhx3 were further identified in the placenta tissue of PE patients and in trophoblast cells cultured under hypoxia. We confirmed that both HOXD8 and Lhx3 positively regulated DIAPH2-AS1 and RP11-445K13.2, resulting in the down-regulation of PAX3. Coincidentally, HOXD8 and Lhx3 also play key roles in tissue development. HOXD8 is involved in the regulation of tissue migration and cell differentiation and organ morphogenesis during embryogenesis [15], while Lhx3 is required for pituitary development and motor neuron specification [16]. A previous study found that HOXD3 is constitutively expressed in the skin of mid-gestational mice, whereas HOXD8 expression was found to be increased in foetal excisional wounds at mid-gestation, suggesting a potential role in scarless wound repair [17]. The mechanism underlying the increased expression of HOXD8 and Lhx3 in the placentas of PE patients and trophoblast cells under hypoxia remains unclear. Their up-regulation is likely due to the stress response under hypoxia. Several lncRNAs, such as MEG3, TUG1, PVT1, and MVIH, have been shown to be involved in the occurrence and development of PE. However, DIAPH2-AS1 and RP11-445K13.2 have seldom been investigated previously, and as a result their physiological and pathological functions are largely unknown. The present study revealed that DIAPH2-AS1 and RP11-445K13.2 mediated the regulatory effects of HOXD8 and Lhx3 in PAX3.

Although all these transcription factors and lncRNAs were found to be involved in the regulation of PAX3 in HTR-8/SVneo cells under hypoxia, the network of HOXD8/DIAPH2-AS1 exerted the most profound effect. Thus, this study focused on how the HOXD8/DIAPH2-AS1 network epigenetically regulates PAX3 expression. The amount of LSD1 and DNMT1 that binds to H3 is notably increased in HTR-8/SVneo cells in response to hypoxia. However, the depletion of both HOXD8 and DIAPH2-AS1 blocked the combination of LSD1 and DNMT1 with H3, which was accompanied by an increased percentage of H3K4me2 in H3 [10]. These data suggest that hypoxia promoted the expression of DIAPH2-AS1 by HOXD8. DIAPH2-AS1 further recruited LSD1 to H3, resulting in a decreased percentage of H3K4me2 in H3. Notably, LSD1 not only regulated the methylation of histones, but also controlled DNA methylation by regulating DNMT1. A previous study found that targeted deletion of the gene encoding LSD1 in embryonic stem cells induces progressive loss of DNA methylation. This is due to the fact that LSD1 demethylates and stabilizes DNMT1. Therefore, DIAPH2-AS1 recruiting LSD1 to H3 would theoretically increase the stability of DNMT1 at H3, which may enhance the function of DNMT1 at the promoter region of *Pax3*.

The present study concludes that the deletion of HOXD8 or DIAPH2-AS1 or the direct increase of Pax3 attenuated the inhibition of the proliferation, migration, and invasion of HTR-8/SVneo cells upon hypoxia. These results suggest that the HOXD8/DIAPH2-AS1/Pax3 network plays an important role in regulating the function of HTR-8/SVneo cells under hypoxia. In summary, the present study determined that this network is responsible for the down-regulation of Pax3 in trophoblast cells, thus improving our understanding of PE pathogenesis.

## Abbreviations

ChIP: chromatin immunoprecipitation
EZH2: enhancer of zeste homolog 2
EVT: extravillous trophoblast
LcRNA: long non-coding RNA
LSD1: lysine-specific demethylase 1
MeDIP: methylated DNA immunoprecipitation
ncRNA: non-coding RNA
PE: pre-eclampsia
RIP: RNA immunoprecipitation
